# Lymphangioleiomyomatosis With Atypical Presentation Following Pneumothorax: A Case Report

**DOI:** 10.7759/cureus.48121

**Published:** 2023-11-01

**Authors:** Rita Q Rodrigues, Margarida M Carvalho, Conceição Souto-Moura, Ana Loureiro

**Affiliations:** 1 Pulmonology Department, Centro Hospitalar Trás-os-Montes e Alto Douro, Vila Real, PRT; 2 Pathology and Laboratory Medicine, Centro Hospitalar Universitário de São João, Porto, PRT

**Keywords:** segmentectomy, pneumothorax, congenital pulmonary airway malformation, pulmonary cysts, lymphangioleiomyomatosis

## Abstract

Lymphangioleiomyomatosis (LAM) is a rare systemic disease that typically presents like cystic lung disease. High-resolution computed tomography (CT) is the recommended imaging technique, with cysts being the hallmark: typically multiple, well-circumscribed, thin-walled, with a variable diameter (usually <2 cm) and widespread in distribution. The gold standard for diagnosis is a biopsy. LAM should be considered in the differential diagnosis of cystic lung diseases.

The authors report a case of LAM presenting with a pneumothorax, which due to its atypical imaging characteristics, mimicked another uncommon cystic disease. A multidisciplinary approach is crucial when dealing with presentations of rare diseases.

## Introduction

Lymphangioleiomyomatosis (LAM) is a rare systemic disease, almost exclusively occurring in women of reproductive age, that typically presents like a cystic lung disease. It is also associated with lymphatic abnormalities (lymphadenopathy and cystic masses of the axial lymphatics) and abdominal angiomyolipomas [1].

Two forms of LAM have been described: the sporadic form and the less common form associated with tuberous sclerosis [2]. LAM is characterized by diffuse lung cysts, usually multiple and smaller than 2 cm, which result from the proliferation of smooth muscle-like cells (LAM cells) [3]. The main manifestations are chronic dyspnea, pneumothorax, and chylous pleural effusion [4]. Pneumothorax is often the first manifestation of the disease and can recur [1,5].

## Case presentation

A 20-year-old female nonsmoker, with passive smoking from her father and no other major exposures, was diagnosed with clinical hyperandrogenism, pectus excavatum, and scoliosis.

She presented at the emergency department with a two-week-long right chest pain, associated with dry cough and dyspnea. Physical examination revealed decreased vesicular breath sounds throughout the right hemithorax on pulmonary auscultation.

**Figure 1:**
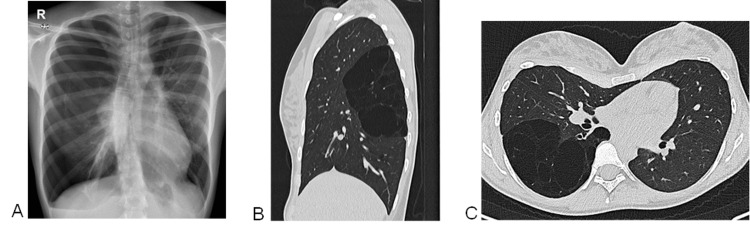
Imaging studies' findings (A) Chest radiography: large right-sided pneumothorax. (B) and (C): Chest CT - (B) sagittal and C) axial. Parenchymal rarefaction with panlobular emphysematous areas and air cysts, in the distribution of the apical segment of the right lower lobe. The right superior segmental lower lobe bronchus shows an apparent reduction in its caliber at its origin and downstream ectasia. Left deviation of mediastinum, conditioned by pectus excavatum.

Chest X-ray revealed a complete pneumothorax on the right hemithorax (Figure 1A). A diagnosis of primary spontaneous pneumothorax led to her admission to the Pulmonology Department. With the placement of a chest drainage catheter, the pneumothorax resolved within two days without complications. Upon further pulmonology consultation, the patient was found to be asymptomatic and had a normal physical examination. Both the autoimmune tests and alpha-1-antitrypsin levels were normal. Chest computed tomography (CT) showed parenchymal rarefaction with panlobular emphysematous areas and air cysts, in the distribution of the upper segment of the right lower lobe. This right lower lobe segmental bronchus displayed an apparent reduction in caliber at its origin and ectasia downstream (Figure 1B and Figure 1C).

Pulmonary function tests (PFT), including carbon monoxide diffusing capacity (DLCO), were within the predicted values. Bronchoscopy was normal, and bronchial aspirate showed no microbiological isolates.

Suspecting a congenital pulmonary airway malformation (CPAM) or bronchial atresia, an atypical resection of right segment six was performed. Histologically, the segmentectomy samples showed dilated bronchial/bronchiolar stroma containing mucus. The walls of these structures and vessels, as well as the cystic spaces, are focally lined with elongated cells (smooth muscle cells) without atypia. The hemosideric pigment was seen on macrophages. HMB45 and Melan-A immunohistochemical staining were negative; actin expression was positive, and beta-catenin was inconclusive.

**Figure 2:**
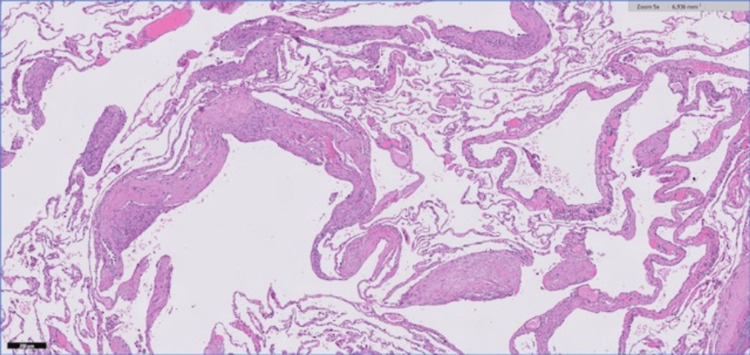
Histology of the segmentectomy sampling Scattered areas of smooth muscle proliferation in the interstitium.

Despite the negativity for HMB45, the morphological aspects observed - cystic spaces with focal smooth muscle cell proliferation, areas of emphysema and hemorrhage, associated with secondary bronchiectasis, areas of fibrosis and dystrophic calcification - were consistent with LAM (Figure 2), according to the referring pathologist. In the multidisciplinary meeting on interstitial lung diseases, the diagnosis of LAM was established. A genetic test of the lung specimen was negative for TSC1 and TSC2, and abdominal CT was normal.

The patient is under medical follow-up every six months with chest CT scans and PFT. She remains asymptomatic and without new episodes of pneumothorax. The chest CT shows architectural disorganization of the right lower lobe due to surgery, without further changes, including the appearance of new cystic lesions. PFT continue within the expected values.

## Discussion

LAM is a rare disease characterized by diffuse lung cysts that result from the proliferation of atypical smooth muscle-like cells - LAM cells. Despite its diffuse distribution, LAM is more closely related to a low-grade destructive neoplasm than to interstitial lung disease [4]. As a standard of diagnosis, lung or lymphatic biopsy is distinguished by two hallmarks: lung cysts and proliferation of LAM cells in airways, veins, and lymphatic vessels, resulting in cystic structures in the axial lymphatics and cystic remodeling of the pulmonary parenchyma [6]. Hemosiderosis is common due to hemorrhage resulting from the rupture of dilated venules [7].

In immunochemistry, melanocytic markers (e.g., HMB45 and Melan-A) and muscle markers (e.g., actin, desmin, and vimentin) are often positive [6].

HMB45 staining is historically associated with LAM, but sometimes it can be absent and it is not required to establish the diagnosis [6].

The main manifestations of LAM are pneumothorax (over 50%), progressive dyspnea (over 70%), and chylothorax (less common) [4,5]. Pneumothorax is frequently the primary condition leading to diagnosis, and recurrence is common [4,5]. Other manifestations include cough, chest pain, hemoptysis, and chyloptysis [1,7].

At presentation, lung function is often normal, but the most common initial abnormality is decreased diffusing capacity for carbon monoxide (DLCO). A progressive airflow obstruction leading to respiratory failure and *cor pulmonale* is present in LAM, but the rate of progression is variable [8].

Chest radiographs are usually normal in the early stages of the disease, although it may present with pneumothorax or pleural effusion [4]. For the diagnosis of LAM, high-resolution CT is recommended; lung cysts are a characteristic feature and are present in most patients [7]. Cysts are characteristically multiple, well-circumscribed, thin-walled, with a variable diameter (usually <2 cm) and ubiquitous distribution [3,4].

Cystic lung diseases, such as pulmonary Langerhans cell histiocytosis, lymphocytic interstitial pneumonia, Birt-Hogg-Dubé syndrome, and emphysema, should be considered as differential diagnoses for LAM [6].

As a congenital cystic lesion of the segmental lung with abnormal bronchial development, CPAM is also a differential diagnosis for large pulmonary cysts, especially in young non-smokers. As the most common congenital lung disease, it typically presents in childhood and rarely in adulthood [3]. Unilobar cysts are more frequent and tend to occur in the lower lobes [9]. Resection is recommended due to reports of malignancies developing from CPAM [3].

## Conclusions

This case reports an LAM with an atypical cystic distribution that, due to its imaging characteristics, mimicked another rare pathology - CPAM with a presentation in adulthood. The patient was referred to surgery earlier due to a high suspicion of CPAM. Additionally, this case highlights the importance of investigating the cause of a pneumothorax, even at a young age, and the need for a multidisciplinary approach to rare disease presentations.
